# Comparison and combined use of NEWS2 and GCS scores in predicting mortality in stroke and traumatic brain injury: a multicenter retrospective study

**DOI:** 10.3389/fneur.2024.1435809

**Published:** 2024-08-06

**Authors:** Wei Hu, Ke Shang, Liqin Chen, Xin Wang, Xia Li

**Affiliations:** ^1^School of Nursing, Jinzhou Medical University, Jinzhou, Liaoning, China; ^2^People's Hospital of Shangrao, Shangrao, China; ^3^Huaian Hospital of Huaian City, Huai’an, China; ^4^First Affiliated Hospital of Jinzhou Medical University, Jinzhou, China

**Keywords:** stroke, brain injuries, traumatic, National Early Warning Score (NEWS2), Glasgow Coma Scale (GCS)

## Abstract

**Objective:**

This study aims to assess the effectiveness of the National Early Warning Score 2 (NEWS2) versus Glasgow Coma Scale (GCS) in predicting hospital mortality among patients with stroke and traumatic brain injury (TBI).

**Location:**

This multicenter study was conducted at two anonymized tertiary care hospitals in distinct climatic regions of China, with a combined annual emergency admission exceeding 10,000 patients.

**Patients:**

The study included 2,276 adult emergency admissions diagnosed with stroke (*n* = 1,088) or TBI (*n* = 1,188) from January 2021 to December 2023, excluding those with chronic pulmonary disease, severe cardiac conditions, or a history of brain surgery.

**Measuring and main outcomes:**

The receiver operating characteristic (ROC) curve and the area under the curve (AUC) were utilized to analyze the predictive accuracy of NEWS2 and GCS for hospital mortality at 24, 48, and 72 h post-admission and at discharge.

**Results:**

Out of 2,276 patients (mean age 61.4, 65.6% male), 1855 survived while 421 succumbed. NEWS2 demonstrated superior predictive accuracy (AUC = 0.962) over GCS (AUC = 0.854) for overall hospital mortality. Specifically, NEWS2 outperformed GCS in predicting mortality at 24 h (0.917 vs. 0.843), 48 h (0.893 vs. 0.803), and 72 h (0.902 vs. 0.763). Notably, despite a higher AUC for NEWS2 at predicting 24-h hospital mortality, the sensitivity and specificity of GCS were considerably lower (12 and 31%, respectively) compared to NEWS2 (sensitivity of 95% and specificity of 81%). Subgroup analysis showed NEWS2 outperforming GCS in predicting in-hospital mortality for TBI and stroke patients. For TBI patients (*n* = 260), NEWS2 had an AUC of 0.960 (95% CI: 0.948–0.973) vs. GCS’s AUC of 0.811 (95% CI: 0.781–0.840). For stroke patients (*n* = 161), NEWS2 had an AUC of 0.930 (95% CI: 0.908–0.952) vs. GCS’s AUC of 0.858 (95% CI, 0.823–0.892). NEWS2 showed greater sensitivity in both groups, highlighting its effectiveness in identifying high-risk neurological patients.

**Conclusion:**

NEWS2 scores are more precise and effective in predicting hospital mortality in stroke and TBI patients compared to GCS scores, although slightly less so within the first 24 h. Combining NEWS2 with GCS and clinical findings within the initial 24 h is recommended for a comprehensive prognosis evaluation.

## Introduction

Stroke and traumatic brain injury are common, acute neurological diseases frequently encountered in emergency departments (1). The timely evaluation of patients’ vital signs and the severity of their conditions is crucial in managing these acute neurological events (2). The Glasgow Coma Scale (GCS) is widely recommended as an objective tool for assessing the degree of consciousness impairment and the trend of disease progression. While the GCS correlates with disease prognosis and mortality rates to some extent (3), it has notable limitations (4). For instance, in early mild traumatic brain injury (TBI), GCS scores may not be sensitive enough, even if the patient already exhibits abnormalities on a CT scan (5). Unlike NEWS 2, GCS focuses specifically on assessing the neurological system. It is a highly specialized tool used to describe the severity of injury and detect neurological deterioration.

The UK’s National Early Warning Score 2 (NEWS 2) (Supplementary Figure S1) consolidates several physiological parameters, including respiratory rate, oxygen saturation, body temperature, systolic pressure, heart rate, and level of consciousness (6), to effectively identify clinical deterioration in hospitalized patients (7). NEWS 2 has been widely adopted globally. It was designed to detect episodes of possible hemodynamic instability, respiratory failure, and dysthermia. Moreover, NEWS 2 includes an assessment of the level of consciousness, which correlates with the verbal component of the GCS scale. As described by the developers of NEWS 2, “If the patient has new-onset confusion, disorientation, and/or agitation, where previously their mental state was normal – this may be subtle. The patient may respond to questions coherently, but there is some confusion, disorientation, and/or agitation. This would score 3 or 4 on the GCS (rather than the normal 5 for verbal response), and scores 3 on the NEWS system” (8). These features make NEWS 2 particularly effective in quickly identifying changes in the condition of hospitalized patients and aiding in triaging those who need immediate medical attention. Although both scoring systems include an assessment of the level of consciousness, unlike NEWS 2, GCS is a strict neurological assessment tool used to evaluate consciousness/coma, describe the degree of injury, and detect neurological deterioration (9). It is important to note that the GCS score is rarely used in isolation for clinical assessment but rather as part of a comprehensive evaluation. Neither score was originally developed to predict mortality, especially since neuroprognostication requires a multimodal approach (10).

This study aims to explore the potential utility of combining the NEWS 2 and GCS scoring systems in predicting mortality among stroke and traumatic brain injury patients, especially in the context of large-scale datasets. Existing studies in this area are relatively limited. Therefore, our research seeks to provide new evidence to support the combined use and further development of the GCS and NEWS 2 scoring systems. Additionally, age is a critical prognostic factor influencing mortality in these patients, yet its impact has not been thoroughly studied within the combined NEWS 2 and GCS scoring context. This study will provide new evidence to support the combined use and development of the GCS and NEWS 2 scoring systems and analyze the effect of age on the prognosis of patients with these acute neurological conditions.

## Methods

In this multicenter retrospective study, we selected patients who were admitted non-electively to two tertiary comprehensive hospitals in China as our study participants. The inclusion criteria were patients who were diagnosed for the first time with either stroke or traumatic brain injury (TBI) and were over 18 years of age. The exclusion criteria included patients with a history of chronic pulmonary disease, serious cardiac disease, or those who had undergone neurosurgery. Following these criteria, a total of 2,276 patients were enrolled in the study. Subgroup analyses were conducted to evaluate the predictive performance of NEWS2 and GCS scores in different patient groups, specifically those with stroke and those with TBI. To report the sensitivity and specificity for the GCS and NEWS2 scores, we used different cutoffs (e.g., GCS ≤8, GCS ≤12, and NEWS2 ≥ 5, NEWS2 ≥ 6, NEWS2 ≥ 7). To optimize the best sensitivity and specificity, we employed the Louden test method. This helped to determine the most suitable cut points for the different scoring systems.

### Baseline data

Vital signs at the time of admission were collected, including but not limited to blood pressure, heart rate, and body temperature.

### Scoring system data

The Glasgow Coma Scale (GCS) and the National Early Warning Score (NEWS2) were documented upon admission.

### Clinical information

This encompassed the patient’s age, gender, body mass index (BMI), medical history (including hypertension, diabetes, hyperlipidemia, etc.), lifestyle habits (such as smoking and alcohol consumption), as well as the duration from onset of symptoms to hospital admission (see Supplementary Table S1 for baseline information).

### Outcome tracking

The condition of the patients was documented at 24, 48, and 72 h post-admission, along with the final in-hospital outcomes (including recovery, transfer, or death) to assess the value of these indicators in predicting mortality rates among patients who suffered from strokes and traumatic brain injuries.

### Statistical analysis

**Figure 1:**
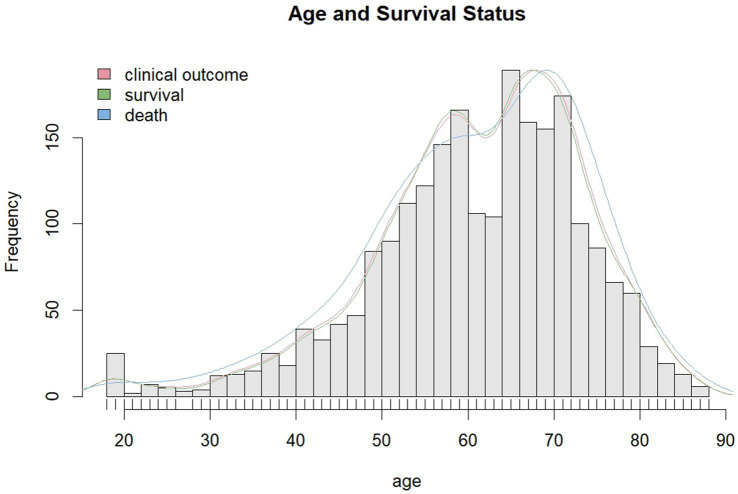
Shows the distribution of the relationship between age and mortality in patients with traumatic brain injury and stroke.

We explored the relationship between age and mortality rate in stroke and traumatic brain injury patients (Figure 1). First, we employed kernel density estimation to plot the kernel density estimation graph of age and death rate. Subsequently, a chi-square test on a 2×2 table was used to compare categorical variables, and a rank-sum test was used for comparing continuous variables. The threshold for establishing statistical significance was a *p*-value less than 0.05. Comparisons were made between stroke and traumatic brain injury patients for differences in NEWS2 and GCS scores, to determine which score could better predict mortality. We therefore plotted the corresponding ROC (Receiver Operating Characteristic) curves for both NEWS2 and GCS scores (9) and calculated their AUC (Area Under the Curve). An AUC value close to 1 indicates high prediction accuracy of the model, while a value close to 0.5 suggests poor predictive performance. A model with an AUC equal to or greater than 0.7 is likely to have good discriminative ability (10). Building on this, we further subdivided the NEWS2 and GCS scores and plotted and calculated the ROC curves and AUC values for in-hospital 24-h, 48-h, 72-h, and final outcome mortality rates under each segmented NEWS2 and GCS score. To compare the performance of different scoring systems in predicting patient mortality risk, we calculated the ROC curves for each scoring system at different cutoffs and used the DeLong test to compare the AUCs of these ROC curves. The DeLong test helped us determine the significance of differences between the AUCs of different scores, thereby confirming whether one scoring system might be superior to another. All data analyses were executed using R language (version 4.3.2) (11), with packages such as pROC and ggplot2 for analysis. This study furnished crucial information for the prognosis evaluation of stroke and traumatic brain injury patients and further proposed specific scoring methods applicable to clinical practice.

## Results

**Figure 2:**
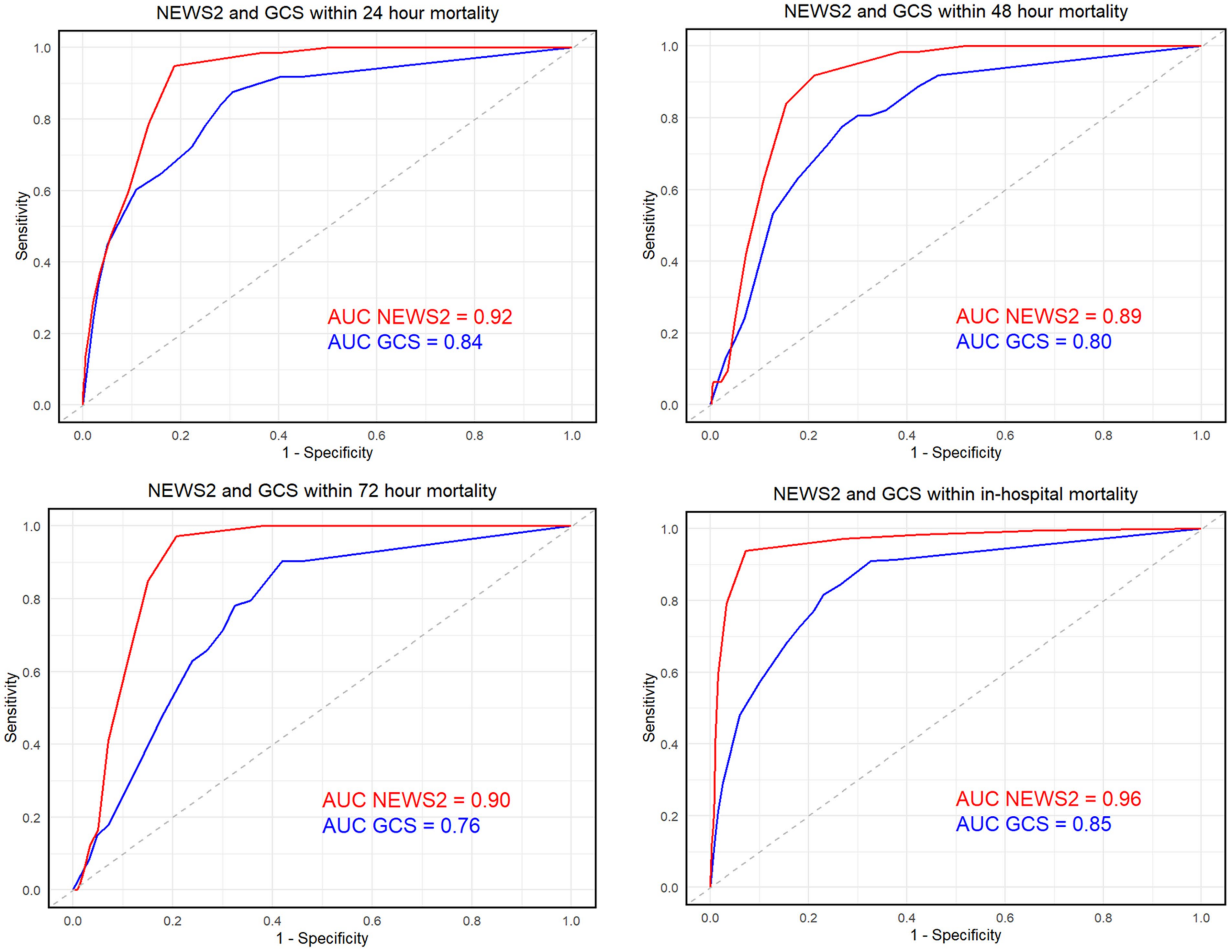
Shows the receiver operating characteristic (ROC) curve and the area under the curve (AUC) for the NEWS score (in black) and the GCS score (in gray) at 24 h, 48 h, 72 h, and the final outcome”.

**Figure 3:**
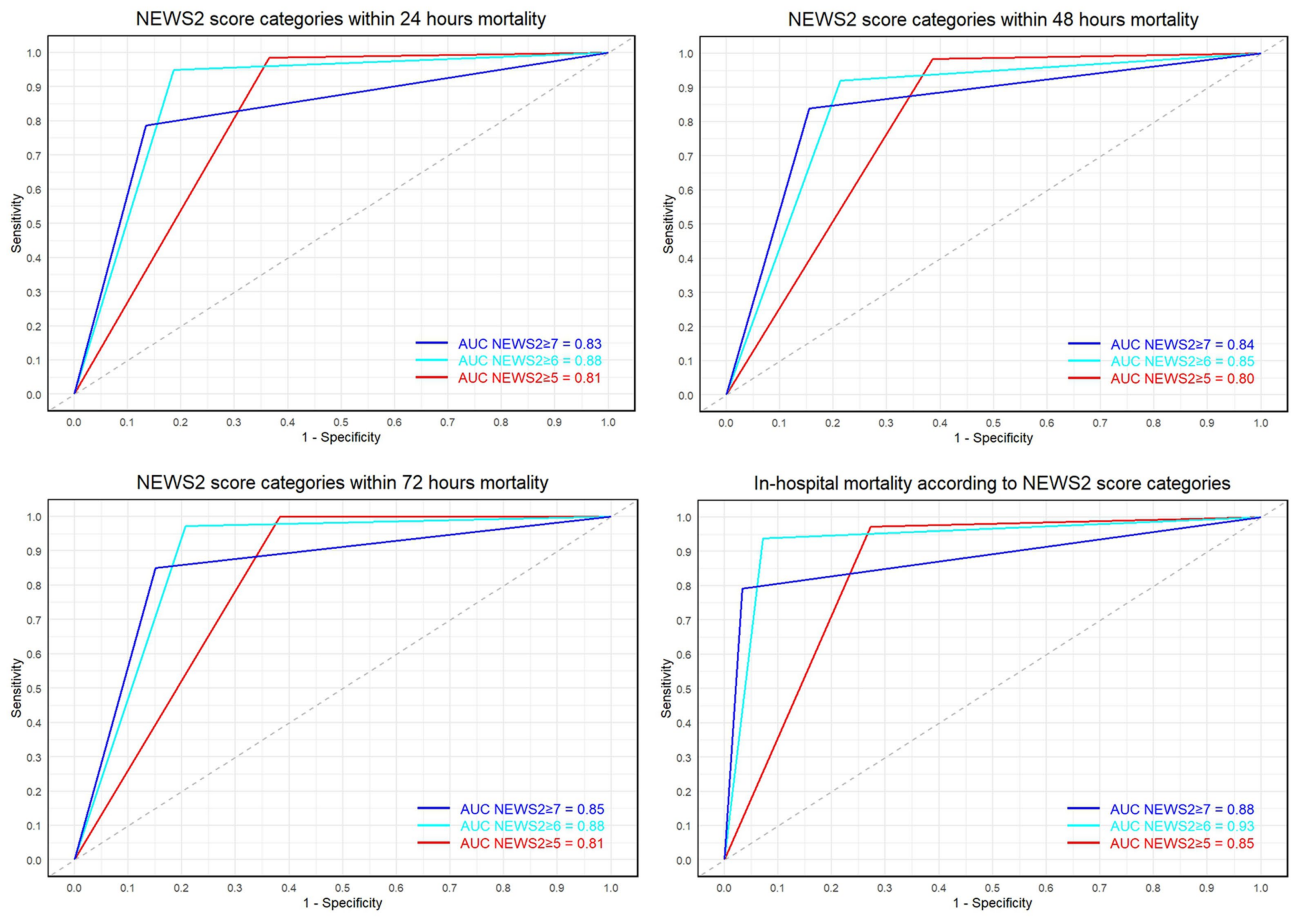
Displays the ROC curves and AUC values for NEWS2 scores ≥5, 6, and 7 at 24 h, 48 h, 72 h, and final outcomes.

In this study, we collected data from the First Affiliated Hospital of Jinzhou Medical University in northern China and the Shangrao Hospital affiliated with Nanchang University in southern China, including a total of 2,276 eligible patients with acute stroke and traumatic brain injury. Their clinical characteristics and observational outcomes are listed in Supplementary Table S1. In the survival group, 62.7% were male, with an average age of 61.5 years, and a median BMI of 23.9. In the death group, 65.6% were male, with an average age of 61.2 years, and a median BMI of 24.0. There were 136 deaths within 24 h, 62 within 48 h, and 73 within 72 h. We illustrated the predictive ability of the NEWS2 and GCS scores for 24-h, 48-h, 72-h, and final mortality outcomes in Table 1. This is quantified as the AUC, sensitivity, specificity, positive predictive value, negative predictive value, as well as positive and negative likelihood ratios, and provided the 95% confidence intervals for each indicator (Figure 2). These integrated results offer a comprehensive prognosis evaluation and assist us in understanding and comparing the predictive performance of the two scoring systems. Additionally, we analyzed the relationship between age and mortality. In the stroke group, the correlation coefficient between age and mortality was 0.00113945, with a *p*-value of 0.9687; in the TBI group, the correlation coefficient was 0.0215257, with a *p*-value of 0.4785515. This indicates that, in our study, there is no significant correlation between age and mortality (see Figure 3).

**Table 1:** Sensitivity analysis of in-hospital mortality risk, 24-hour mortality risk, 48-hour mortality risk, and 72-hour mortality risk based on NEWS2 and GCS scores.

Mortally death	Sensitivity% (95%CI)	Specificity% (95%CI)	PPV (%) (95%CI)	NPV (%) (95%CI)	LR+ (95%CI)	LR− (95%CI)	AUC (95%CI)
In-hospital NEWS2 421	94 (91,96)	93 (92,94)	75 (71,78)	99 (98,99)	13.83 (11.17,15.32)	0.04 (0.02,0.07)	0.962 (0.952,0.972)
In-hospital GCS 421	18 (15,22)	23 (21,25)	5 (4,6)	55 (52,59)	0.24 (0.2,0.29)	3.54 (3.28,3.82)	0.854 (0.834,0.874)
Within 24 hours NEWS2 136	95 (90,98)	81 (80,83)	24 (21,28)	100 (99,100)	5.08 (4.69,5.51)	0.06 (0.03,0.13)	0.917 (0.901,0.932)
Within 24 hours GCS 136	12 (7,19)	31 (29,33)	1 (1,2)	85 (82,87)	0.18 (0.12,0.27)	2.87 (2.71,3.04)	0.843 (0.809,0.878)
Within 48 hours NEWS2 62	92 (82,97)	79 (77,80)	11 (8,14)	100 (99,100)	4.32 (4.01,4.65)	0.1 (0.05,0.23)	0.893 (0.870,0.916)
Within 48 hours GCS 62	23 (13,35)	27 (25,29)	1 (0,1)	92 (90,94)	0.31 (0.21,0.46)	2.89 (2.65,3.16)	0.803 (0.754,0.853)
Within 72 hours NEWS2 73	97 (90,100)	79 (77,81)	13 (11,17)	100 (100,100)	4.69 (4.36,5.04)	0.03 (0.01,0.13)	0.902(0.885,0.919)
Within 72 hours GCS 73	10 (4,19)	42 (40,44)	1 (0,1)	93 (92,95)	0.17 (0.08,0.32)	2.15 (2.06,2.25)	0.763 (0.717,0.810)
In-hospital (TBI group) NEWS2 260	70 (64,76)	32 (28,34)	19 (16,21)	30 (21,39)	0.73 (0.70,0.76)	7.47 (5.2,10.74)	0.960 (0.948,0.973)
In-hospital (TBI group) GCS 260	25 (19,30)	29 (26,32)	10 (8,12)	55 (50,60)	0.35 (0.29,0.42)	2.59 (2.36,2.86)	0.811 (0.781,0.840)
In-hospital (stroke group) NEWS2 161	74 (66,80)	20 (10,30)	10 (8,12)	32% (21,46)	0.75 (0.71,0.79)	14.35 (8.73.23.59)	0.930 (0.908,0.952)
In-hospital (stroke group) GCS 161	18 (12,25)	18 (16,20)	3 (2,4)	60 (54,65)	0.22 (0.16,0.29)	4.61(4.07.5.20)	0.858(0.823,0.892)

NEWS2, National Early Warning Score 2; GCS, Glasgow Coma Scale.

Upon integrating the results of Table 1, the NEWS2 score far exceeds the GCS score in accurately, stably, and reliably predicting in-hospital mortality. Its sensitivity and specificity both reach 94 and 93%, demonstrating an impressive level of accuracy even with few misdiagnoses, and its dynamic risk identification ability is commendable. In contrast, the sensitivity and specificity of the GCS score are only 18 and 23% respectively, with significant shortcomings in the accuracy of case classification. The positive predictive value (PPV) and negative predictive value (NPV) of the NEWS2 score reach 75 and 99% respectively, with very high accuracy in predicting in-hospital mortality and survival, whereas the PPV and NPV of the GCS score are only 5 and 55% respectively, with significantly reduced accuracy. Furthermore, the positive likelihood ratio (LR+) of the NEWS2 score reaches 13.8, whereas its negative likelihood ratio (LR-) is only 0.04, indicating a distinct predictive advantage. On the other hand, the positive and negative likelihood ratios of the GCS score are only 0.24 and 3.54 respectively, significantly diminishing the reliability of death outcome predictions.

According to the 24-h, 48-h, and 72-h data, the AUC indicators of NEWS2 scores were 0.917, 0.893, and 0.902, respectively, presenting an excellent and stable predictive performance overall. Especially in terms of sensitivity and NPV, the values were close to or equal to 100%, demonstrating extremely high predictive accuracy. In contrast, the GCS score showed its AUC values as 0.843, 0.803, and 0.763 respectively, significantly lower than NEWS2, and both its sensitivity and specificity were also lower than NEWS2, indicating that its predictive accuracy and stability were unsatisfactory.

We compared the ROC curves for three different NEWS2 thresholds (≥5, ≥6, and ≥ 7). The optimal sensitivity and specificity for NEWS2 ≥ 5 (threshold 0.5) were: Sensitivity: 97.1%, Specificity: 72.7%. For NEWS2 ≥ 6 (threshold 0.5), they were: Sensitivity: 93.8%, Specificity: 92.8%. For NEWS2 ≥ 7 (threshold 0.5), they were: Sensitivity: 79.1%, Specificity: 96.7%. To compare the AUC of these thresholds’ ROC curves, we performed the DeLong test with the following results: For the comparison between NEWS2 ≥ 5 and NEWS2 ≥ 6: *Z* value: −13.142, *p* value: < 0.05, 95% Confidence Interval: −0.09648830 to −0.07144417. For the comparison between NEWS2 ≥ 5 and NEWS2 ≥ 7: *Z* value: −2.8027, *p* < 0.05, 95% Confidence Interval: −0.050553422 to −0.008945587. For the comparison between NEWS2 ≥ 6 and NEWS2 ≥ 7: *Z* value: 6.0697, *p* < 0.05, 95% Confidence Interval: 0.03670971 to 0.07172374.

For the NEWS2 scores, a threshold of ≥5 was observed to have high sensitivity (97.1%) but lower specificity (72.7%). Adjusting the threshold to ≥6 resulted in a slight decrease in sensitivity (93.8%) but a significant increase in specificity (92.8%). When the threshold was set to ≥7, sensitivity further decreased (79.1%), but specificity reached the highest (96.7%). The AUC values for the three thresholds were 0.8493, 0.9333, and 0.8790, respectively.

According to the DeLong test results, NEWS2 ≥ 6 had a significantly higher AUC compared to NEWS2 ≥ 5 (Z = −13.142, *p* < 0.05) and NEWS2 ≥ 7 (Z = 6.0697, *p* < 0.05). This indicates that the performance of NEWS2 ≥ 6 in distinguishing survival from death outcomes is optimal. Compared to NEWS2 ≥ 5 and NEWS2 ≥ 7, NEWS2 ≥ 6 improves the identification of high-risk patients, albeit with a slight loss in sensitivity.

Table 2 our results indicate that with a NEWS2 score ≥ 6, the scoring system demonstrates high sensitivity (94%) and specificity (93%) in predicting patient mortality risk, superior to settings of NEWS2 scores ≥5 or ≥ 7. Although the positive likelihood ratio (LR+) peaks at NEWS2 score ≥ 7 (24.04), signifying a strong indicator of severe patient conditions, the sensitivity decreases at this threshold, potentially leading to missed cases of actual mortality. Over time, there is a discernible downward trend in each assessment metric; however, overall, the NEWS2 score remains a reliable predictive marker.

**Table 2:** Sensitivity analysis of in-hospital mortality risk, 24-hour mortality risk, 48-hour mortality risk, and 72-hour mortality risk, based on NEWS2 scores ≥5, NEWS2 scores ≥6, NEWS2 scores ≥7, GCS scores ≤8, and GCS scores ≤12.

Mortally death	Sensitivity% (95%CI)	Specificity% (95%CI)	PPV (%) (95%CI)	NPV (%) (95%CI)	LR+ (95%CI)	LR− (95%CI)	AUC (95%CI)
In-hospital							
NEWS2≥5 409	97 (95,97)	73 (71,75)	45 (41,48)	99 (98,100)	3.55 (3.34,3,79)	0.04 (0.02,0.07)	0.849 (0.836.0.862)
NEWS2≥6 395	94 (91,96)	93 (92,94)	75 (71,78)	99 (98,99)	13.08 (11.17,15.32)	0.07 (0.05,0.1)	0.933 (0.92,0.946)
NEWS2≥7 333	79 (75,83)	97 (81,88)	85 (81,88)	95 (94,96)	24.04 (18.84,30.68)	0.22 (0.18,0.25)	0.879 (0.859,0.899)
Within 24 hours							
NEWS2≥5 134	99 (95,100)	63 (61,66)	15 (12,17)	100 (99,100)	2.7(2.58,2.82)	0.02 (0.01,0.09)	0.81 (0.796,0.824)
NEWS2≥6 129	95 (90,98)	81 (80,83)	24 (21,28)	100 (99,100)	5.08 (4.69,5.51)	0.06 (0.03,0.13)	0.881 (0.861,0.901)
NEWS2≥7 107	79 (71,85)	87 (85,88)	27 (23,32)	98 (98,99)	5.86 (5.26,6.53)	0.25 (0.18,0.33)	0.826 (0.791,0.862)
Within 48 hours							
NEWS2≥5 61	98 (91,100)	61 (59,63)	7 (5,8)	100 (100,100)	2.55 (2.45,2.66)	0.03 (0.00,0.18)	0.799 (0.780,0.818)
NEWS2≥6 57	92 (82,97)	79 (77,80)	11 (8,14)	100 (99,100)	4.32 (4.01,4.65)	0.1 (0.05,0.23)	0.853 (0.818,0.888)
NEWS2≥7 52	84 (72,92)	85 (83,86)	13 (10,17)	99 (99,100)	5.43 (4.91,6)	0.19 (0.11,0.32)	0.842 (0.795,0.889)
Within 72 hours							
NEWS2≥5 73	100 (95,100)	62 (60,64)	8 (6,10)	100 (100,100)	2.62 (2.51,2.73)	0.001 (0.001,0.002)	0.890 (0.799,0.819)
NEWS2≥6 71	97 (90,100)	79 (77,81)	13 (11,17)	100 (100,100)	4.69 (4.36,5.04)	0.03 (0.01,0.13)	0.883 (0.862,0.903)
NEWS2≥7 72	85 (75,92)	85 (83,86)	16 (12,20)	99 (99,100)	5.63 (5.10,6.22)	0.18 (0.11,0.29)	0.894 (0.807,0.891)
In-Hospital							
GCS≤8 287	68 (63,73)	85 (83,86)	50 (46,54)	92 (91,93)	4.4 (3.97,4.89)	0.38 (0.34,0.42)	0.763 (0.74,0.787)
GCS≤12 355	84 (80,88)	74 (72,76)	42 (39,45)	95 (94,96)	3.2 (2.99,3.42)	0.21 (0.17,0.26)	0.790 (0.77,0.81)
Within 24 hours							
GCS≤8 98	72 (64,79)	78 (76,79)	17 (14,20)	98 (97,98)	3.24 (2.96,3.54)	0.36 (0.29,0.45)	0.794 (0.71,0.788)
GCS≤12 121	89 (82,94)	66 (64,68)	14 (12,17)	99 (98,99)	2.63 (2.50,2.77)	0.17 (0.11,0.26)	0.776 (0.784,0.804)
Within 48 hours							
GCS≤8 45	73 (60,83)	76 (74,78)	8 (6,10)	99 (98,99)	3.04 (2.74,3.37)	0.36 (0.26,0.51)	0.743 (0.687,0.800)
GCS≤12 51	82 (70,91)	64 (62,66)	6 (5,8)	99 (99,100)	2.3 (2.15,2.45)	0.28 (0.17,0.45)	0.732 (0.683,0.781)
Within 72 hours							
GCS≤8 46	63 (51,74)	76 (74,78)	8 (6,11)	98 (98,99)	2.63 (2.32,2.98)	0.49 (0.38,0.62)	0.695 (0.639,0.752)
GCS≤12 58	79 (68,88)	64 (62,66)	7 (5,9)	99 (98,99)	2.23 (2.08,2.39)	0.32 (0.21,0.48)	0.719 (0.671,0.767)

NEWS2, National Early Warning Score 2; GCS, Glasgow Coma Scale.

We compared ROC curves for two different GCS thresholds (≤8 and ≤ 12). For GCS ≤8 (threshold 0.5), the optimal sensitivity and specificity were: sensitivity: 68%, specificity: 85%. For GCS ≤12 (threshold 0.5), the optimal sensitivity and specificity were: sensitivity: 84%, specificity: 74%. To compare the AUC of the ROC curves between GCS ≤8 and GCS ≤12, we performed a DeLong Test with the following results: *Z* value: −5.0818, *p* < 0.05, 95% Confidence Interval: −0.07854745 to −0.03482237. According to the DeLong Test results, the AUC for GCS ≤12 was significantly higher than that for GCS ≤8 (*p* < 0.05), indicating better performance in distinguishing between survival and death outcomes for GCS ≤12. Adjusting the GCS threshold to ≤8 resulted in a sensitivity of 68% and a specificity of 85%. When the threshold was adjusted to ≤12, sensitivity increased to 84%, but specificity slightly decreased to 74%. The AUC values for the two thresholds were 0.763 and 0.790 respectively, showing similar overall performance of the GCS scores in predicting mortality risk, although GCS ≤12 improves the identification of high-risk patients.

**Figure 4:**
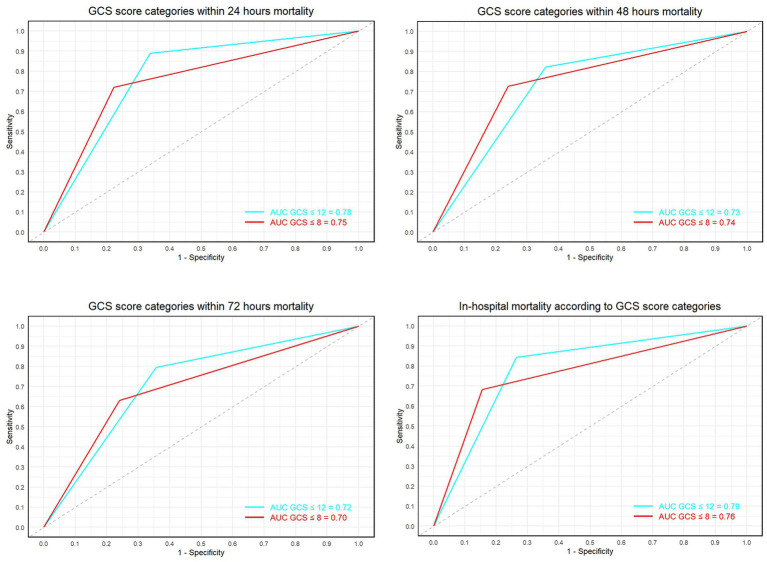
Shows the ROC curves and AUC values for GCS scores ≤8 and ≤ 12 at 24 h, 48 h, 72 h, and final outcomes.

For the GCS scores, a threshold of ≤8 was observed to have lower sensitivity (68%) but higher specificity (85%). Adjusting the GCS threshold to ≤12 resulted in an increased sensitivity (84%), albeit with a slight reduction in specificity (74%) (12). The AUC values under both thresholds (0.763 and 0.790) (Figure 4) indicate similar overall performance of the GCS score in predicting mortality risk, though adjusting the score improves identification of high-risk patients.

Subgroup analysis revealed that NEWS2 significantly outperformed GCS in predicting in-hospital mortality for both TBI and stroke patients. For TBI patients (*n* = 260), NEWS2 had an AUC of 0.960 (95% CI: 0.948–0.973) compared to GCS’s AUC of 0.811 (95% CI: 0.781–0.840). The sensitivity and specificity of NEWS2 in the TBI group were 70% (95% CI: 64–76) and 32% (95% CI: 28–34), respectively, with a PPV of 19% (95% CI: 16–21) and an NPV of 30% (95% CI: 21–39). The LR+ was 0.73 (95% CI: 0.70–0.76) and the LR- was 7.47 (95% CI: 5.2–10.74). In contrast, GCS had a sensitivity of 25% (95% CI: 19–30) and specificity of 29% (95% CI: 26–32), with a PPV of 10% (95% CI: 8–12) and an NPV of 55% (95% CI: 50–60). The LR+ was 0.35 (95% CI: 0.29–0.42) and the LR- was 2.59 (95% CI: 2.36–2.86).

For stroke patients (*n* = 161), NEWS2 had an AUC of 0.930 (95% CI: 0.908–0.952) compared to GCS’s AUC of 0.858 (95% CI: 0.823–0.892). The sensitivity and specificity of NEWS2 in the stroke group were 74% (95% CI: 66–80) and 20% (95% CI: 10–30), respectively, with a PPV of 10% (95% CI: 8–12) and an NPV of 32% (95% CI: 21–46). The LR+ was 0.75 (95% CI: 0.71–0.79) and the LR- was 14.35 (95% CI: 8.73–23.59). In contrast, GCS had a sensitivity of 18% (95% CI: 12–25) and specificity of 18% (95% CI: 16–20), with a PPV of 3% (95% CI: 2–4) and an NPV of 60% (95% CI: 54–65). The LR+ was 0.22 (95% CI: 0.16–0.29) and the LR- was 4.61 (95% CI: 4.07–5.20). ROC curves for these analyses are provided in Supplementary Figure S2, and detailed data can be found in Table 1.

This study connected hospital data from the north and the south, systematically investigating the clinical characteristics and observed outcomes of 2,276 acute stroke and traumatic brain injury patients. Among the numerous scoring systems, we specifically evaluated the predictive abilities of the NEWS2 and GCS scoring systems for patient outcomes at 24 h, 48 h, 72 h, and final fatality. The results show that the NEWS2 scoring system is superior to the GCS score in the accuracy, stability, and reliability of predicting in-hospital mortality. Through data analysis, we found that in the GCS scores, GCS ≤ 8 has a higher specificity (85%) and a relatively good positive predictive value (PPV of 50%), and a higher negative predictive value (NPV of 92%) at the final outcome, with an AUC value of 0.763, demonstrating its high specificity and reasonable predictive efficacy at a lower scoring threshold for severe prognosis. This finding solves the problem of prognosis assessment for patients with acute stroke and traumatic brain injury in clinical practice, providing an important clinical decision tool for practical diagnosis (13).

On this basis, we believe that the NEWS2 scoring system (especially when the score is ≥6) can more accurately predict the patient’s risk of death (14), making it more clinically valuable compared to the GCS scoring system. This not only reinforces the advantage of the NEWS2 scoring system in predicting the mortality risk of acute stroke and traumatic brain injury patients, but also reveals that the GCS ≤ 8 scoring threshold, as an auxiliary tool, has practical value under certain conditions for in-depth assessment of patient risk.

The GCS score and NEWS2 scoring system can be used in combination to provide a more comprehensive and accurate clinical assessment. The GCS score is more accurate in assessing the function of the nervous system (15); for patients with obvious vital sign changes in acute conditions or systemic diseases, the NEWS2 score has more advantages (16). If the two scores are inconsistent, further detailed evaluation of the patient is needed. Overall, combining these two scoring systems not only enhances the accuracy of judgment on patient’s clinical status, but also helps with early detection of critical cases and taking timely interventions, providing more comprehensive, more personalized diagnosis and treatment care plans (17, 18). In this regard, we propose the combined application of the GCS score and NEWS2 scoring system in clinical practice to improve the prognosis quality of patients with acute stroke and traumatic brain injury (19, 20).

In this study, we explored the independent value of the GCS and NEWS2 scoring systems in predicting in-hospital mortality, but we did not evaluate their combined application in the actual data. We recommend that future research design specialized combined assessment models to further validate the value of their joint application. This approach will not only enhance the accuracy of assessing the clinical status of patients but also aid in the early identification of critical cases and timely interventions, providing more comprehensive and personalized diagnostic and treatment plans.

Despite the significant results obtained, some limitations that need to be addressed. First, this study only covers patients from two hospitals, so there may be regional biases. In the future, the adaptability of the study can be improved by including data from more regions and hospitals. Second, in our biochemical value prediction analysis at early admittance, we did not take into account some congenital diseases and complications, such as liver and kidney dysfunction, abnormal blood lipids, etc., which may also affect the accuracy of biochemical values. Therefore, future studies can further explore the impact of these factors on the predictive model (21). In general, this study conducted a deep and comprehensive comparative analysis of the NEWS2 and GCS scoring systems in predicting hospital mortality for patients with acute stroke and traumatic brain injury, providing empirical evidence for the clinical diagnosis of these patients. We expect future studies to improve based on this work, incorporating more accurate and comprehensive prediction models into clinical practice, to further improve the treatment effects and prognosis quality of patients (22).

## Conclusion

This study utilized data from 2,276 patients across two hospitals in China, enhancing the generalizability of the results. In terms of comparative analysis, the study evaluated the predictive efficacy of the National Early Warning Score 2 (NEWS2) versus the Glasgow Coma Scale (GCS), providing valuable insights that aid clinical decision-making. Additionally, detailed statistical assessments were conducted, including analyses of AUC, sensitivity, specificity, PPV, NPV, and likelihood ratios, to comprehensively evaluate the predictive performance of each scoring system. The results indicated that NEWS2 significantly outperformed GCS in terms of predictive accuracy and stability, demonstrating higher sensitivity and specificity. The study also recommends the combined use of NEWS2 and GCS to potentially enhance patient care and prediction accuracy, thereby offering practical clinical applications and improvements. Furthermore, specific analyses were conducted for patients with traumatic brain injury (TBI) and stroke, providing detailed insights into the unique predictive outcomes for each subgroup.

The study has several limitations. The sample was restricted to two hospitals in China, potentially introducing regional bias and limiting the generalizability of the results. The study only assessed the individual predictive value of NEWS2 and GCS, without evaluating their combined application, missing the opportunity for a comprehensive prediction model. Future research should include data from more diverse regions and hospitals to validate and enhance the adaptability of these scoring systems. It is important to emphasize how NEWS2 (particularly scores ≥6) can significantly improve the early identification of high-risk patients and outcomes through timely interventions. Although GCS can effectively identify patients with poor prognosis in certain situations (e.g., scores ≤12), its overall predictive capacity for in-hospital mortality is limited. While GCS accurately assesses neurological impairment, as a standalone prognostic tool, it fails to capture the dynamic changes of acute disease and lacks other critical clinical parameters essential for predicting overall health status. Future research should explore the combined use of NEWS2 and GCS in clinical practice to provide a more comprehensive and accurate risk assessment for patients.

## Data availability statement

The raw data supporting the conclusions of this article will be made available by the authors, without undue reservation.

## Ethics statement

The studies involving humans were approved by First Affiliated Hospital of Jinzhou Medical University and People’s Hospital of Shangrao. The studies were conducted in accordance with the local legislation and institutional requirements. Written informed consent for participation was not required from the participants or the participants’ legal guardians/next of kin in accordance with the national legislation and institutional requirements.

## Author contributions

WH: Data curation, Formal analysis, Investigation, Software, Validation, Visualization, Writing – original draft. KS: Investigation, Writing – review & editing, Methodology. LC: Data curation, Investigation, Writing – review & editing. XW: Data curation, Investigation, Writing – review & editing. XL: Funding acquisition, Methodology, Project administration, Writing – review & editing.
