# Supplementary figures and images for: Comparison and combined use of NEWS2 and GCS scores in predicting mortality in stroke and traumatic brain injury: a multicenter retrospective study
Source: Front Neurol. 2024 Aug 6;15:1435809. doi: 10.3389/fneur.2024.1435809 (PMC11333856; doi:10.3389/fneur.2024.1435809)

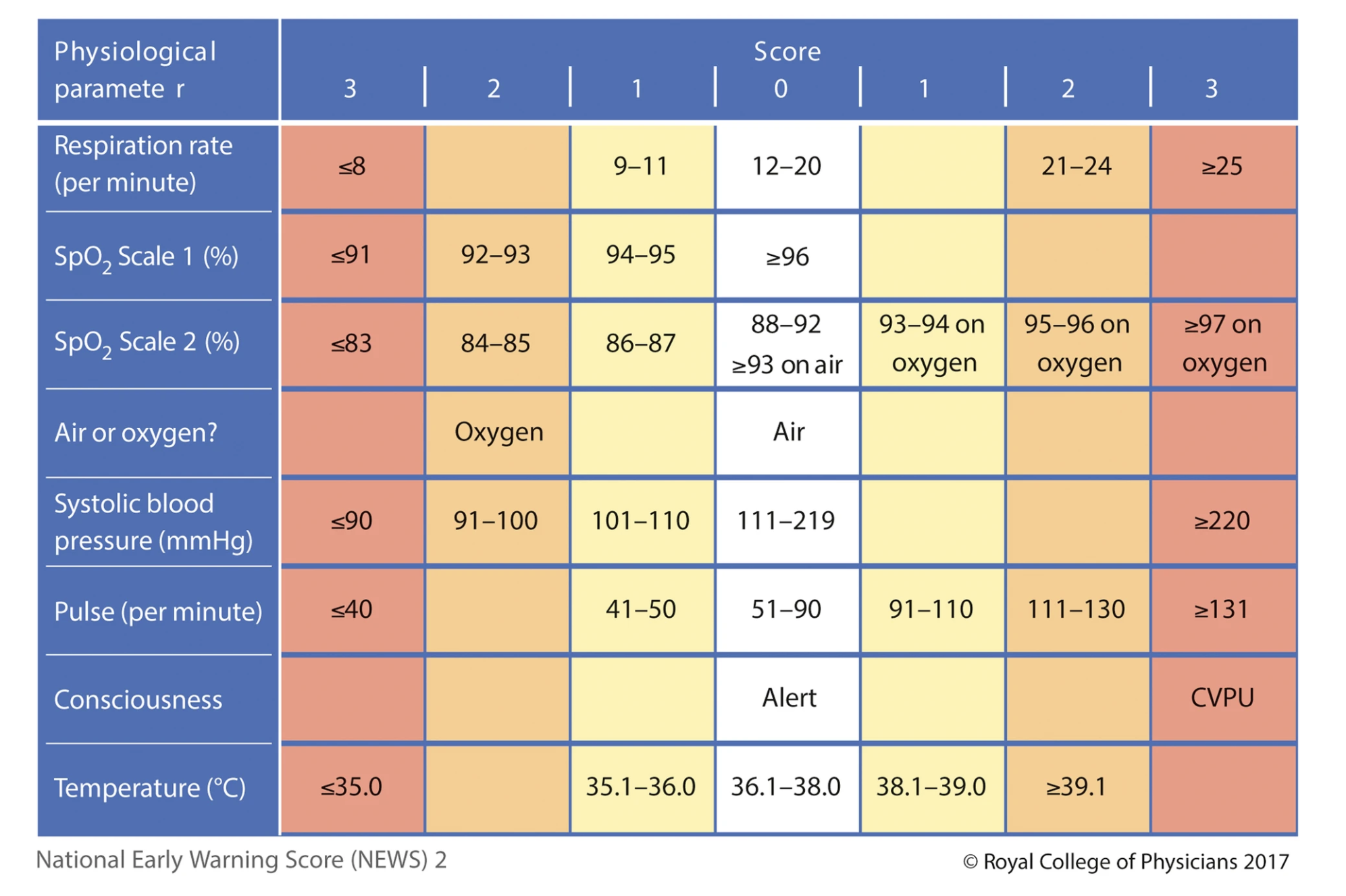

Supplement: Supplementary file 1 [file Image_1.TIF]

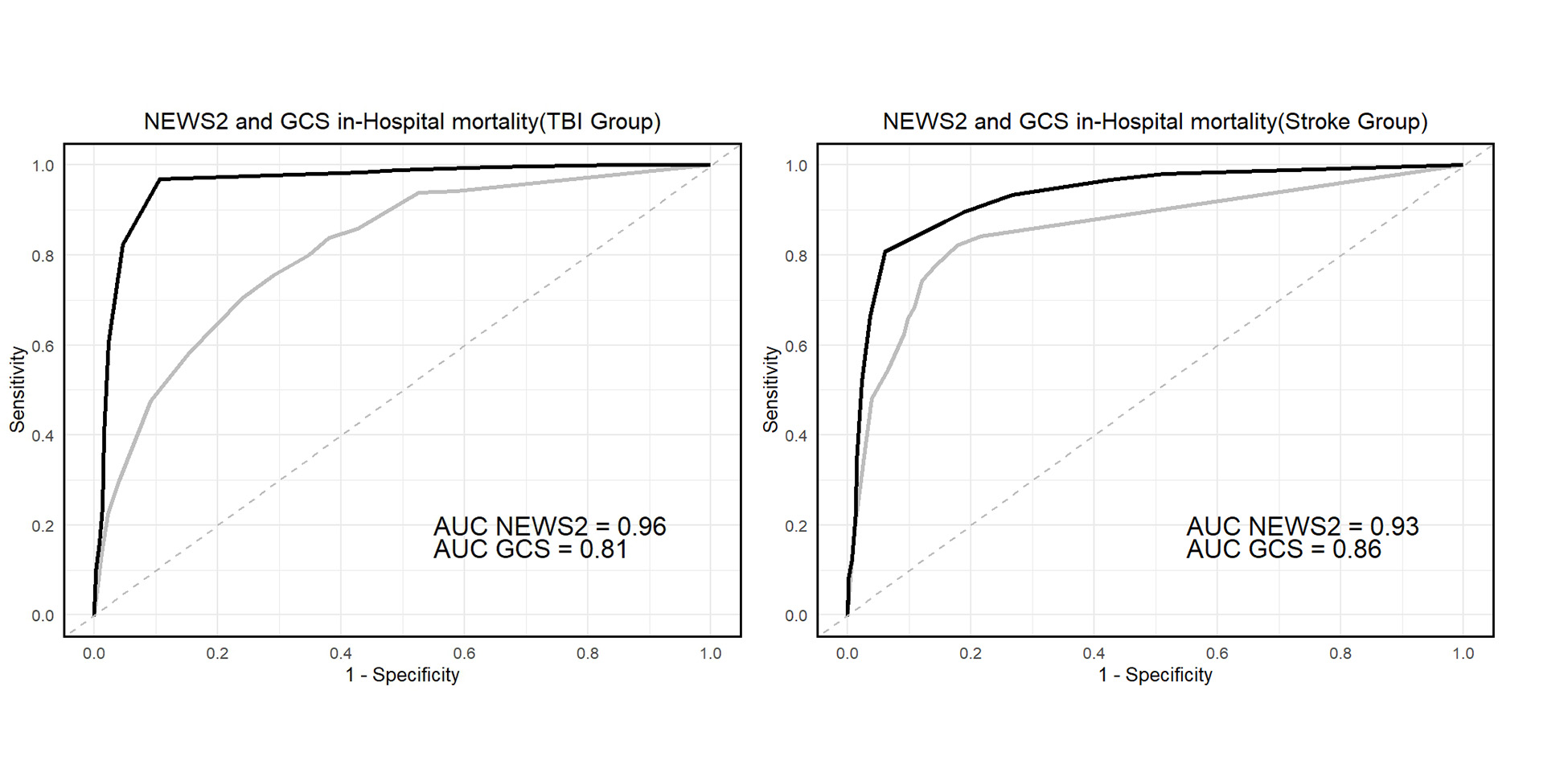

Supplement: Supplementary file 2 [file Image_2.JPEG]
