# Supplementary material for: Comparison and combined use of NEWS2 and GCS scores in predicting mortality in stroke and traumatic brain injury: a multicenter retrospective study
Source: Front Neurol. 2024 Aug 6;15:1435809. doi: 10.3389/fneur.2024.1435809 (PMC11333856; doi:10.3389/fneur.2024.1435809)
Supplement: Supplementary file 3 [file Table_1.DOCX]

| **Supplemental Table 1 Neurological signs upon emergency admission for patients with cerebrovascular accident (stroke) and traumatic brain injury** | | | |
| --- | --- | --- | --- |
| Characteristic | Survive | Death | P- value |
| N | 1855 | 421 | **-** |
| Male (%)  Stroke(%)  TBI(%) | 1164（62.7）  927（85.2）  928（78.1） | 276（65.6）  161（14.8）  260（21.9） | 0.28  ＜0.01  ＜0.01 |
| Mean age (years) (SD) | 61.5（12.2） | 61.2（12.8） | 0.70 |
| BMI（IQR）*(kg/m2) | 23.9（4.9） | 24.0（4.86） | 0.27 |
| Smoking（%） | 496（26.7） | 122（29） | 0.35 |
| drinking alcohol(%) | 505 (27.2) | 125 (29.7) | 0.31 |
| fecal and urinary incontinence l(%) | 243 (13.1) | 36 (47.7) | ＜0.01 |
| rural population（%） | 1365（73.6） | 282（67） | 0.006 |
| history of chronic disease（%） |  |  | 0.139 |
| normal | 839（45.2） | 188（45） |  |
| diabetes | 135（7.3） | 33（7.3） |  |
| hypertension | 740（39.9） | 181（43） |  |
| HTN-DM | 121（6.5） | 14（3.3） |  |
| Vital signs |  |  |  |
| Temperature(%) |  |  | ＜0.01 |
| 36.1-38.0℃ | 1846（99.5） | 398（94.5） |  |
| 38.1-39.0℃ | 0 | 5（2.1） |  |
| 35.1-36.0℃ | 36（0.3） | 8（2） |  |
| ≤35.0℃ | 3（0.2） | 10（2.4） |  |
| Respiratory rate(breaths per minute（%） |  |  | ＜0.01 |
| 12-18 | 1767(95.3) | 256(61) |  |
| ≥25 | 14（0.8） | 56（13.3） |  |
| 21-24 | 71（4） | 103（24.5） |  |
| 9-11 | 0 | 4（1） |  |
| ≤8 | 0 | 1（0.2） |  |
| Oxygen saturation（%） |  |  | ＜0.01 |
| ≥96% | 1748（94.2） | 152（36.1） |  |
| 94%-95% | 71（3.8） | 154（36.6） |  |
| 92%-93% | 23（1.2） | 42（1） |  |
| ≤91% | 13（0.7） | 73（17.3） |  |
| Air or oxygen(%) |  |  | ＜0.01 |
| Air or Device | 1031(55.6) | 67(15.9) |  |
| O_2_ | 823(44.4) | 354(84.1) |  |
| Blood Pressure mmHg(%) |  |  | ＜0.01 |
| 111-219 | 1781（96.0） | 290（68.9） |  |
| ≥220 | 37（2.0） | 87（20.7） |  |
| 101-110 | 18（1.0） | 7（1.7） |  |
| **Supplemental Table 1 Neurological signs upon emergency admission for patients with cerebrovascular accident (stroke) and traumatic brain injury** | | | |
| 91-100 | 14（0.8） | 10（2.4） |  |
| 51-90 | 3（0.2） | 24（5.7） |  |
| ≤50 | 1（0.1） | 3（0.7） |  |
| Pulse Beats/min(%) |  |  | ＜0.01 |
| 51-90 | 1589（85.7） | 174（41.3） |  |
| ≥130 | 9（0.5） | 38（9） |  |
| 111-130 | 28（1.5） | 51（12.1） |  |
| 91-110 | 221（11.9） | 146（34.7） |  |
| 41-50 | 7（0.4） | 8（1.9） |  |
| 31-40 | 1（0.1） | 4（1） |  |
| Consciousness（%） |  |  | ＜0.01 |
| Alert | 1174（63.2） | 35（8.3） |  |
| Confusion | 4（0.2） | 0 |  |
| Voice | 219（11.8） | 41（9.7） |  |
| Pain | 186（10） | 58（13.8） |  |
| Unconscious | 271（14.6） | 287（68.2） |  |
| Mean Bun (SD) | 5.82（2.96 | 7.48（4.26） | ＜0.01 |
| Mean Cr (SD) | 70（45.7） | 88.7（70.1） | ＜0.01 |
| Mean ALB (SD) | 38.4（4.47） | 36.3（5.68） | ＜0.01 |
| Mean KCL (SD) | 3.9（0.447） | 3.83（0.631） | ＜0.01 |
| Mean TP (SD) | 66.8（6.99） | 64.9（9.75） | ＜0.01 |
| Mean AST (SD) | 21.8（18.2） | 41.1（40.6） | ＜0.01 |
| Mean GCS (SD) | 13.0（3.15） | 7.64（3.64） | ＜0.01 |
| GCS≤8 score（%） | 287（15.5） | 287（68.2） | ＜0.01 |
| GCS≤12 score（%） | 489（26.4） | 355（84.3） | ＜0.01 |
| Mean NEWS2（SD） | 2.45（2.32） | 8.33（2.54） | ＜0.01 |
| NEWS2≥5score（%） | 506（27.3） | 409（97.2） | ＜0.01 |
| NEWS2≥6score（%） | 133（7.2） | 395（93.8） | ＜0.01 |
| NEWS2≥7score（%） | 61（3.3） | 333（79.1） | 0.009 |

Neurological signs upon emergency admission for patients with cerebrovascular accident (stroke) and traumatic brain injury

**Supplemental Table 1:**BMI is missing 1006(54.2%) for Survive and 255(60.6%) for Death，

HTN-DM，coexistence of hypertension and diabetes; BMI, body mass index; NEWS2, National Early Warning Score 2; GCS，Glasgow Coma Scale；Bun: Blood Urea Nitrogen；Cr: Creatinine；ALB: Albumin；KCL: Potassium Chloride；TP: Total Protein；AST: Aspartate Aminotransferase
